# The prevalence and predictive factors of nocturnal polyuria in Japanese patients with nocturia: a multicentral retrospective cohort study

**DOI:** 10.1038/s41598-023-45311-z

**Published:** 2023-10-24

**Authors:** Koji Iinuma, Yoshinori Nishino, Kanako Matsuoka, Tatsuya Ihara, Shunta Makabe, Ryo Tanji, Yuki Harigane, Kenichiro Ishida, Masayoshi Tamaki, Shigeaki Yokoi, Hiroki Hoshino, Kazuya Yuhara, Toru Yamada, Yasuaki Kubota, Kousei Miwa, Mina Kikuchi, Yoshiyuki Kojima, Takahiko Mitsui, Takuya Koie

**Affiliations:** 1 Department of Urology, Graduate School of Medicine, Gifu University, 1-1 Yanagido, Gifu, Gifu 501-1194 Japan; 2 Nishino Clinic, 1-55-2 Mitsuicho, Kagamihara, Gifu 504-0941 Japan; 3 Department of Urology, Fukushima Medical University, 1 Hikariga-Oka, Fukushima, Fukushima 960-1295 Japan; 4 Department of Urology, University of Yamanashi Graduate School of Medical Sciences, 1110 Shimokato, Chuo, Yamanashi 408-3898 Japan; 5 Department of Urology, Ohara General Hospital, 6-1 Uwamachi, Fukushima, Fukushima 960-8611 Japan; 6 Department of Urology, Japanese Red Cross Fukushima Hospital, 7-7 Yashimacho, Fukushima, Fukushima 960-8530 Japan; 7 Department of Urology, Ohta Nishinouchi Hospital, 2-5-20 Nishinouchi, Koriyama, Fukushima 963-8558 Japan; 8 Department of Urology, Gifu Prefectural General Medical Center, 4-6-1 Noisiki, Gifu, Gifu 500-8717 Japan; 9 Department of Urology, Gifu Municipal Hospital, 7-1 Kashimacho, Gifu, Gifu 500-8513 Japan; 10 Department of Urology, Central Japan International Medical Center, 1-1 Kenkonomachi, Minokamo, Gifu 5058510 Japan; 11 Department of Urology, Ogaki Municipal Hospital, 4-86 Minaminokawacho, Ogaki, Gifu 503-8502 Japan; 12 Department of Urology, Japanese Red Cross Takayama Hospital, 3-11 Tenmancho, Takayama, Gifu 506-8550 Japan; 13 Department of Urology, Tokai Central Hospital, 4-6-2 Soharahigashijimacho, Kagamihara, Gifu 504-8601 Japan; 14 Department of Urology, Toyota Memorial Hospital, 1-1 Heiwamachi, Toyota, Aichi 471-0821 Japan; 15 Department of Urology, Japanese Red Cross Gifu Hospital, 3-36 Iwakuracho, Gifu, Gifu 502-8511 Japan; 16 Sugo Clinic, 1-10-16 Sugo, Gifu, Gifu 502-0914 Japan

**Keywords:** Urology, Urogenital diseases

## Abstract

The aims of this study were to determine the prevalence and predictors of nocturnal polyuria (NP) in Japanese patients. This multicentral, observational study enrolled patients with the chief complaint of nocturia at 17 Japanese institutions between January 2018 and December 2022. The frequency of daily voiding and volume of urination were evaluated using bladder diaries. NP was diagnosed in patients with an NP index of > 33%. The primary endpoint was NP prevalence in patients with nocturia. The secondary endpoints were the prevalence of NP according to sex and age and the identification of factors predicting NP. This study analyzed 875 eligible patients. NP was present in 590 (67.4%) patients, with prevalence rates of 66.6% and 70.0% in men and women, respectively. Age ≥ 78 years, body mass index (BMI) < 23.0 kg/m^2^, and patients with ischemic heart or cerebrovascular disease were significant predictors of NP (*P* < 0.001, *P* < 0.001, *P* = 0.014, *P* = 0.016, respectively). This is the first large multicenter study to investigate the prevalence of NP in Japanese patients with nocturia. NP has a prevalence of 67.4%. Significant predictors of NP include age, BMI, and cardiovascular disease.

## Introduction

Nocturia is defined as the number of times during the main sleep period that the patient must be awake for the desire to urinate^1^. In addition, the patient must be asleep or willing to go to sleep each time the patient urinates after the first time of awakening to urinate^1^. It is one of the most common lower urinary tract symptoms (LUTS), and its prevalence does not vary by sex but increases with age^2, 3^. Nocturia has been reported to be a risk factor for sexual dysfunction, depression, and metabolic syndrome^4–6^. In addition, frequent nocturia causing multiple awakenings has been reported to cause sleep deprivation and poor sleep quality, resulting in a significant reduction in the patients’ quality of life (QoL) and interference with daily life^7^. In older adult patients, nocturia has been shown to be a risk factor for falls and fractures, leading to decreased patient QoL and worse prognoses^8^.

In Japan, the population of people aged over 65 years accounted for 28.4% of the total population in 2019. By 2030, the number of Japanese people aged over 75 years is expected to reach 22 million, a phenomenon that no other country in the world has ever experienced^9^. Therefore, nocturia appears to be a critical problem for Japan, as it is facing a super-aging society.

Nocturia can be classified according to its four main causes: (1) 24-h polyuria (excessive urination during the daytime and nighttime); (2) bladder storage disorders, such as overactive bladder (OAB) or benign prostatic hyperplasia (BPH); (3) nocturnal polyuria (NP); (4) various related factors, including OAB and NP^10^.

NP is considered one of the main causes of nocturia and is associated with the decreased secretion of the antidiuretic hormone, arginine vasopressin (AVP)^11, 12^. The treatment for NP has dramatically changed with randomized controlled trials that have shown that desmopressin acetate, a synthetic AVP analog, is an effective and well-tolerated treatment for NP regardless of sex^11, 13, 14^. Accordingly, an accurate diagnosis of the etiology of NP is important for appropriate treatment, necessitating the investigation of the prevalence of NP in patients with nocturia.

Using bladder diaries, the prevalence of NP has been reported to range from 76 to 88% in Europe, the United States, and Canada^11^; however, few studies have clarified the prevalence of NP in Japanese patients with nocturia^4, 8^. Therefore, we conducted a multicenter retrospective study to investigate the prevalence of NP in patients with nocturia and investigated the predictive factors of NP.

## Materials and methods

### Patients

Medical Research Ethics Committee of Gifu University, University of Yamanashi, and Fukushima Medical University approved this study (approval number: 2021-053, 2481, and 2021-154, respectively). Informed consent was not required because of the retrospective nature of the study. Moreover, in accordance with the Japanese Ethics Committee and its ethical guidelines, written informed consent was not obtained because retrospective and observational studies using existing materials and other data have already been published. Instead, we used an opt-out approach and provided patients with the opportunity to decline participation.　More information on this study, which is available only in Japanese, can be found at https://www.med.gifu-u.ac.jp/visitors/disclosure/docs/2021-053.pdf (accessed May 31, 2021). All methods were carried out in accordance with relevant guidelines and regulations and in accordance with the World Medical Association Declaration of Helsinki.

In this multicenter, retrospective, observational study, patients with the chief complaint of nocturia, who visited 17 institutions in Japan between January 2018 and December 2022, were enrolled. Patients with bladder stone disease, urinary tract infections, urogenital malignancies, or those using desmopressin were excluded from the study. The following clinical data were collected in this study: age, sex, body mass index (BMI), Eastern Cooperative Oncology Group performance status (ECOG-PS)^15^, post-void residual, estimated glomerular filtration rate (eGFR), medical history, and concomitant medications. Prostate volume and prostate-specific antigen levels were measured in male patients. Urinary frequency and voided volume were evaluated using a bladder diary, and patients were requested to maintain the bladder diary for at least 2 days^2^. The time and volume of each voiding session, bedtime (intention to sleep), and awakening time were recorded in a diary. After the patients returned their diaries, the mean value of the data was calculated. Clinical variables, such as the number of voiding episodes during the daytime, nighttime, and 24-h periods, as well as the volume of urine during the daytime and nighttime, were obtained from the bladder diaries. Data from the first urination after awakening was recorded as daytime urinary frequency and nighttime urine volume.

### Definition of NP

The NP index (NPi) was used to evaluate NP. NPi was calculated by dividing the nocturnal urine volume by the 24-h urine volume. Patients with an NPi > 33% were diagnosed with NP^2^.

### Statistical analysis

The primary endpoint was the prevalence of NP in patients with nocturia. The secondary endpoints were the prevalence of NP according to sex and age. In addition, logistic regression analysis was used to evaluate the predictors of NP. Data analysis was performed using JMP 14 software (SAS Institute Inc., Cary, NC, USA). The cutoff values for clinical parameters were determined using receiver operating characteristic curve analysis^16^. Statistical significance was defined as a two-sided *P*-value of < 0.05.

## Results

### Patient characteristics

A total of 909 patients with nocturia were enrolled. After applying the exclusion criteria, 875 patients were eligible for analysis. The patient characteristics are listed in Table 1. Thirty-four patients (3.9%) had an ECOG-PS ≥ 2. The most common medical history was hypertension, with 47.3% of the patients receiving antihypertensive medication.

**Table 1:** Patient characteristics.

Number	875
Age (year, median, interquartile range)	75.0 (69.0–80.0)
Gender (number, %)	
Male	655 (74.9)
Female	220 (25.1)
Body mass index (kg/m^2^, median, interquartile range)	23.1 (21.4–25.2)
The Eastern Cooperative Oncology Group performance status (number, %)	
0	717 (81.9)
1	74 (8.5)
2	33 (3.8)
3	1 (0.1)
Unknown	50 (5.7)
Prostate volume (mL, median, interquartile range)	30.0 (20.0–44.0)
Post-void residual (mL, median, interquartile range)	16.0 (0–46.0)
Prostate specific antigen (ng/mL, median, interquartile range)	1.6 (0.9–3.1)
Estimated glomerular filtration rate (mL/min/1.73 m^2^, median, interquartile range)	62.9 (52.0–73.0)
Medical history (number, %)	
Hypertension	518 (59.2)
Dyslipidemia	264 (30.2)
Diabetes mellitus	221 (25.3)
Ischemic heart disease	123 (14.1)
Insomnia	122 (13.9)
Arrhythmia	122 (13.9)
Cerebrovascular disease	115 (13.1)
Spinal disease	89 (10.2)
Chronic obstructive pulmonary disease	56 (6.4)
Dementia	38 (4.3)
Sleep apnea syndrome	30 (3.4)
Concomitant medications (number, %)	
Antihypertensive	414 (47.3)
Alpha-1 blocker1	318 (36.3)
Beta 3-adrenoceptor agonist	184 (21.0)
Diuretic	80 (9.1)
Anticholinergics	78 (8.9)
Phosphodiesterase 5 inhibitor	71 (8.1)

The median age was 75 years, and male patients was 74.9%. The median body mass index was 23.1 kg/m^2^. Patients with the Eastern Cooperative Oncology Group performance status (PS) 0 accounted for 81.9%, while those with PS 2 ≤ were 3.9%. The median values of prostate volume, post-void residual, prostate specific antigen, and estimated glomerular filtration rate were 30.0 mL, 16.0 mL, 1.6 ng/mL, and 62.9 mL/min/1.73 m^2^, respectively. The most prevalent medical history was hypertension, followed by dyslipidemia and diabetes. Among the concomitant medications, antihypertensive were the most common, accounting for 47.3%

### Bladder diary

The median number of daytime and nighttime urinations was 7.6 (interquartile range [IQR]: 6–9) and 3.0 (IQR: 2–4), respectively. Median 24-h, daytime, and nighttime voided volume were 1650 mL (IQR: 1270–2150), 980 mL (IQR: 700–1323), and 630 mL (IQR: 430–860), respectively. The median NPi was 38.0% (IQR: 29.0–49.0).

### The prevalence of NP in patients with nocturia

**Figure 1:**
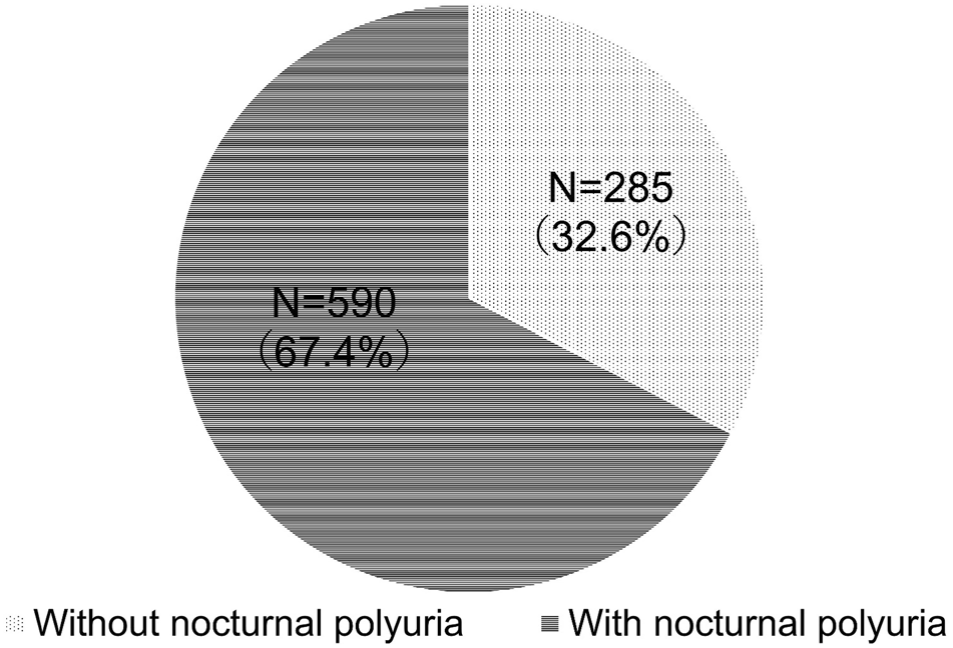
The prevalence of nocturnal polyuria (NP) among enrolled patients with nocturia. Out of 875 patients with nocturia, 590 (67.4%) patients had NP.

**Figure 2:**
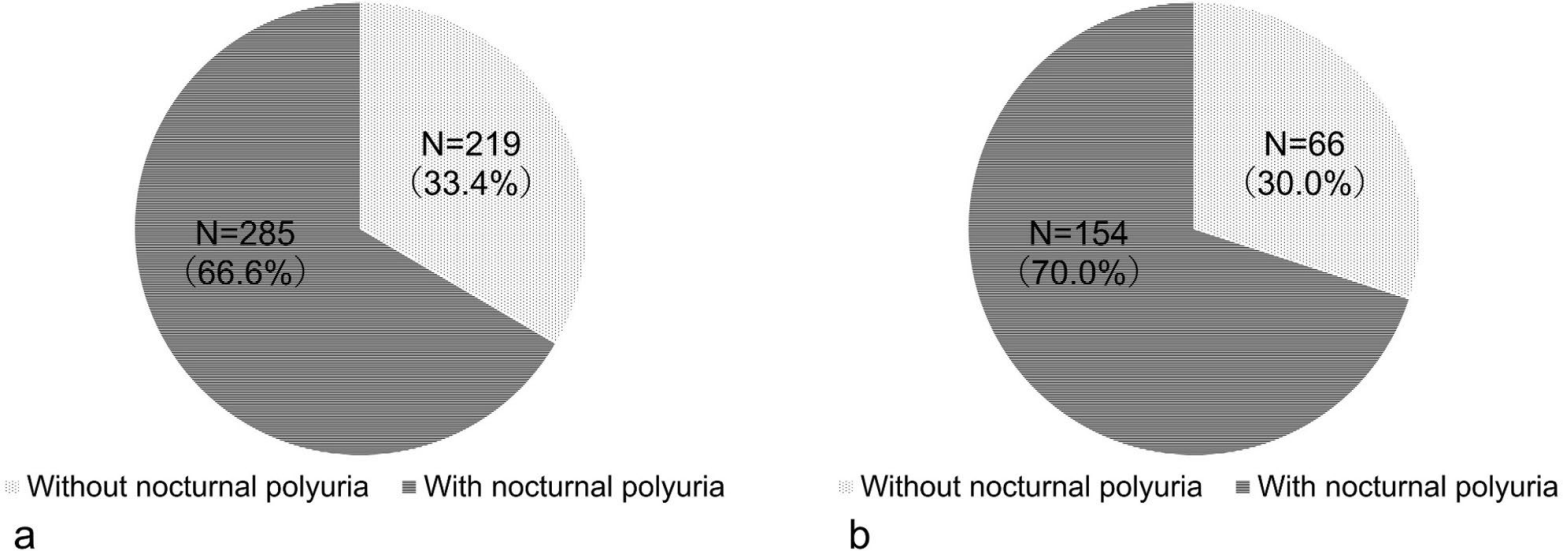
The prevalence of nocturnal polyuria (NP) according to sex in patients with nocturia. (**a**) The prevalence of NP in male patients was 66.6%. (**b**) The prevalence of NP in female patients was 70.0%.

**Figure 3:**
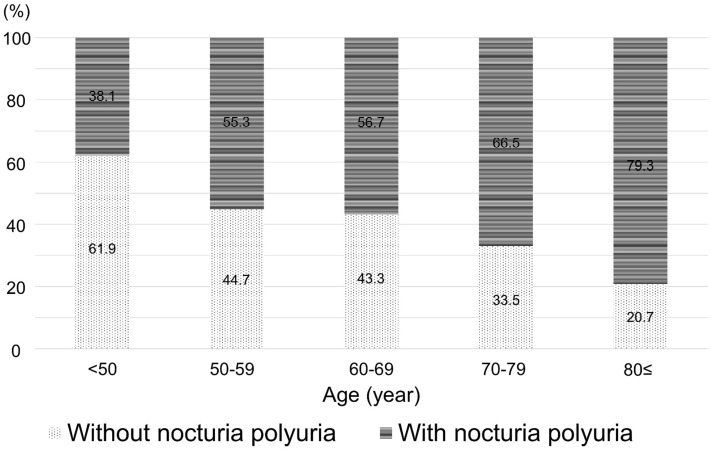
The prevalence of nocturnal polyuria (NP) according to age in patients with nocturia. When patients were divided according to age, the prevalence of NP in patients < 50 years, 50–59 years, 60–69 years, 70–79 years, and 80 ≤ years were 38.1%, 55.3%, 56.7%, 66.5%, and 79.3%, respectively. When examined by age, the prevalence of NP tended to increase with advancing age.

Approximately 70% of the enrolled patients had NP (Fig. 1), and a similar trend was observed when the data was examined according to sex (Fig. 2). When examined by age, the prevalence of NP tended to increase with advancing age (Fig. 3).

### Predictive factors of NP

**Figure 4:**
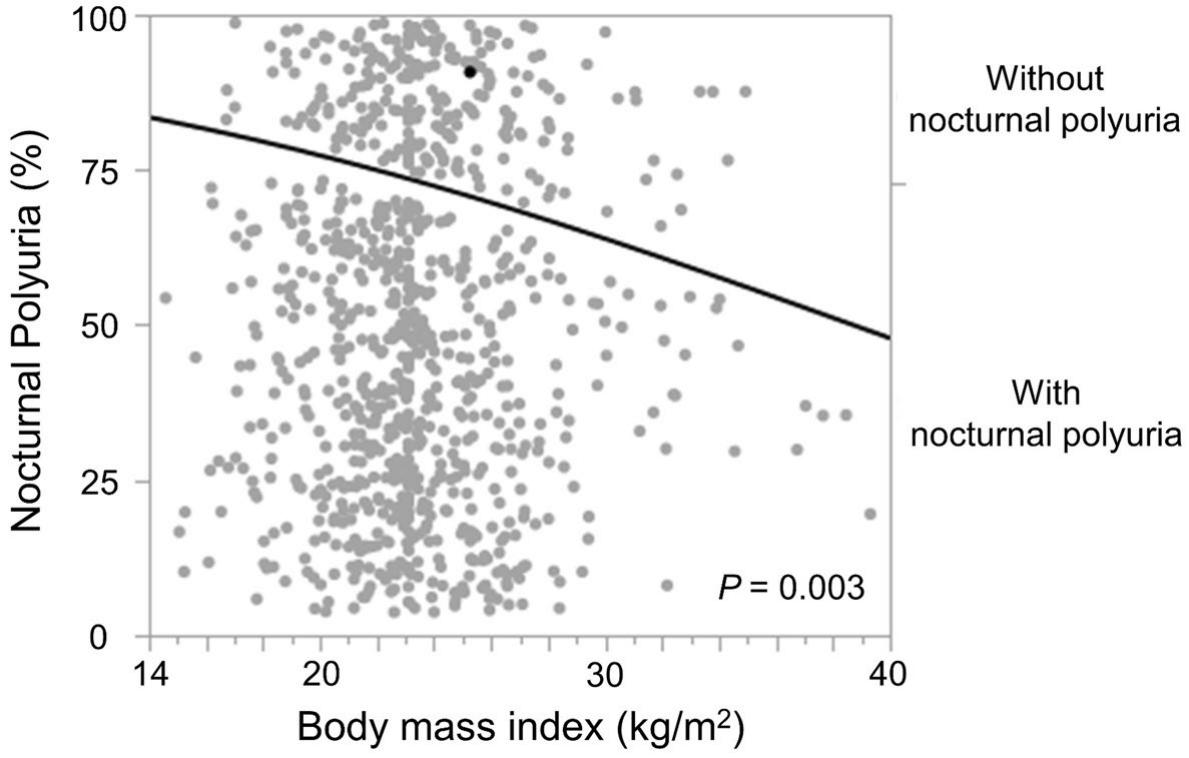
Relationship between body mass index (BMI) and prevalence of nocturnal polyuria (NP); the prevalence of NP significantly decreased with increasing BMI (*P* = 0.003).

The associations between NP and its clinical covariates are presented in Table 2. In the univariate analysis, age, BMI, eGFR, and the presence of hypertension, diabetes, ischemic heart disease, insomnia, and cerebrovascular disease were significant predictors of NP. Conversely, age, BMI, and the presence of ischemic heart and cerebrovascular diseases were significant predictors of NP in the multivariate analysis. Additionally, we evaluated the relationship between BMI and NP in Fig. 4. The prevalence of NP decreased significantly with increasing BMI (Fig. 4).

**Table 2:** Factors evaluated as predictors of nocturnal polyuria.

	n	Univariate analysis			Multivariate analysis		
		HR	95% CI	*P*	HR	95% CI	*P*
Age							
< 78	523	1 (ref.)	–	–	1 (ref.)	–	–
≥ 78	352	2.30	1.69–3.13	< 0.001	1.97	1.43–2.72	< 0.001
Gender							
Male	655	1 (ref.)	–	–	1 (ref.)	–	–
Female	220	1.17	0.84–1.63	0.347	1.17	0.82–1.65	0.383
Body mass index							
< 23.0 kg/m^2^	397	1 (ref.)	–	–	1 (ref.)	–	–
≥ 23.0 kg/m^2^	478	0.57	0.43–0.76	< 0.001	0.57	0.42–0.77	< 0.001
Estimated glomerular filtration rate							
< 45 mL/min/1.73 m^2^	127	1 (ref.)	–	–	1 (ref.)	–	–
≥ 45 mL/min/1.73 m^2^	478	0.63	0.41–0.97	0.035	0.95	0.60–1.52	0.836
Hypertension							
No	357	1 (ref.)	–	–	1 (ref.)	–	–
Yes	518	1.49	1.12–1.99	0.006	1.22	0.89–1.68	0.213
Dyslipidemia							
No	611	1 (ref.)	–	–	1 (ref.)	–	–
Yes	264	1.13	0.83–1.55	0.433	0.92	0.65–1.30	0.626
Diabetes mellitus							
No	654	1 (ref.)	–	–	1 (ref.)	–	–
Yes	221	1.49	1.06–2.10	0.021	1.42	0.98–2.04	0.064
Ischemic heart disease							
No	752	1 (ref.)	–	–	1 (ref.)	–	–
Yes	123	1.96	1.24–3.10	0.004	1.85	1.14–3.01	0.014
Insomnia							
No	753	1 (ref.)	–	–	1 (ref.)	–	–
Yes	122	1.65	1.06–2.57	0.027	1.32	0.83–2.12	0.239
Arrhythmia							
No	753	1 (ref.)	–	–	1 (ref.)	–	–
Yes	122	1.42	0.93–2.19	0.108	1.11	0.71–1.76	0.643
Cerebrovascular disease							
No	760	1 (ref.)	–	–	1 (ref.)	–	–
Yes	115	2.24	1.37–3.64	0.001	1.88	1.13–3.13	0.016
Spinal disease							
No	786	1 (ref.)	–	–	1 (ref.)	–	–
Yes	89	1.43	0.87–2.36	0.155	1.31	0.78–2.19	0.314

In the univariate analysis, age ≥ 78, body mass index (BMI) < 23.0 kg/m^2^, estimated glomerular filtration rate (eGFR) < 45 mL/min/1.73 m^2^, and the presence of hypertension, diabetes, ischemic heart disease, insomnia, and cerebrovascular disease were significant predictors of nocturnal polyuria (NP). Conversely, age ≥ 78, BMI < 23.0 kg/m^2^, and the presence of ischemic heart and cerebrovascular diseases were significant predictors of NP in the multivariate analysis.

*CI* confidence interval, *HR* hazard ratio, *n* number, *ref*. reference.

## Discussion

Several studies have evaluated the prevalence of NP in patients with nocturia^11, 13, 14, 17–19^. Weiss et al.^11^ investigated the prevalence of NP using data from two double-blind, randomized, placebo-controlled phase III trials^13, 14, 17^. The NOCTUPUS trial, which investigated the safety and efficacy of desmopressin for nocturia was conducted primarily in Europe, while another trial that evaluated the effect of desmopressin on nocturia was conducted in North America. In the NOCTUPUS study, the overall prevalence of NP in patients with evaluable frequency-volume chart (FVC) records was 76%, of which 79% of men and 72% of women had NP^13, 14^. In the North American phase III study, the overall prevalence of NP based on FVC was 88%, of which 90% of men and 85% of women had NP^17^. A previous study that analyzed 200 consecutive records of patients complaining of nocturia found that the etiology of nocturia was BPH in 23% of men, urinary incontinence in 4%, stress urinary incontinence in 13%, and detrusor instability in 27%^18^. Overall, only 7% of the cases were due to NP alone, 57% were due to OAB, and 36% were due to both NP and OAB^18^. In contrast, in a study conducted by Chang et al. that investigated the causes of nocturia in 41 male patients, NP was found in 82.9% of the patients and was a significant factor influencing nocturia^19^. In the present study, NP was found in 67.4% of all cases, with a prevalence of 66.6% in male patients and 70.0% in female patients, although the prevalence of NP tended to be slightly lower than that reported in previous studies^12, 18, 19^, NP was found in more than two-thirds of cases regardless of sex, suggesting that NP is one of the most common causes of nocturia in Japanese patients. Despite the essential need for an accurate diagnosis that is dependent on etiology to treat nocturia, the condition is still considered as a storage symptom associated with OAB or BPH and thus has not been adequately treated^19^. Therefore, it is important to use bladder diaries as simple and effective tools to identify patients with NP as the primary cause of nocturia.

In this current study, the prevalence of NP increased with advancing age; a significant predictor of NP was age ≥ 78 years. Various reasons have been postulated for the increase in nocturnal urine volume with age^13, 20–22^. Robertson et al.^20^ reported that the proportion of nocturnal urine volume increased to 34% ± 15% in older individuals compared to approximately 14% ± 4% of the total daily urine volume in those aged 21–35 years. The increase in nocturnal urine volume with age may be due to age-related physiological changes in renal function and circadian rhythm changes in water and electrolyte regulatory hormones^21^. Indeed, decreased renal concentrating capacity and sodium conservation have been observed in the older adult population^21^. Asplund et al.^13^ found that nocturnal serum antidiuretic hormone concentrations were higher in younger adult subjects and lower in older adult subjects^13^. Therefore, changes in water/electrolyte-regulating hormones are thought to contribute to increased nocturnal urine volume^13^. Atrial natriuretic peptide (ANP) is also thought to contribute to increased nocturnal urinary output^22, 23^. Although some reports have found no difference in ANP levels between patients with and without NP^22, 23^, it has been shown that nocturnal ANP levels are increased in patients with NP and that urinary ANP levels are higher in older adult patients with nocturia and NP^22, 23^. These results suggest that physiological changes in the kidneys and related hormonal changes may be associated with the increased prevalence of NP with age.

Interestingly, a BMI < 23.0 kg/m^2^ was a significant predictive factor for NP in this study. Although few studies have examined the association between NP and BMI, several have examined the association between LUTS, nocturia and BMI^24–26^. While some reports have indicated that a high BMI is a risk factor for LUTS and nocturia^24, 25^, both high and low BMI (< 18.5 kg/m^2^) in women are associated with nocturia^26^. In recent years, frailty has received increased attention in the older adult population because of its associated increased risk of falls, disability, hospitalization, and death^27^. The phenotypic definition of frailty comprises five physical characteristics including unintentional weight loss^27^. In fact, a significant correlation between nocturia (four or more episodes), frailty, and polypharmacy has been reported (*P* < 0.05)^28^. Because nocturia is fairly common and is associated with insomnia, frailty, polypharmacy, incontinence, falls, slow walking, and functionality in older adult women, it has been suggested that nocturia is crucial to geriatric practice and that two or more episodes of nocturia could be an indicator of poor health in older adult women^28^. In this study, we examined the relationship between BMI and NP because BMI < 23.0 kg/m^2^ was a significant predictor of NP according to multivariate analysis. Based on the results, we found that the prevalence of NP decreased with increasing BMI. Although the clear reasons for these results remain uncertain, the relationship, including that with frailty, will need to be reconsidered in future prospective studies.

In this study, ischemic heart and cerebrovascular diseases was an independent predictor of NP. In a previous report, nocturia was independently associated with cardiovascular disease after adjusting for other known confounders^29^. Although nocturia was also associated with a lower odds ratio for the presence of heart failure, a history of coronary artery disease was not associated with nocturia^29^. The association between nocturia and cardiovascular morbidities raises several interesting questions. Nocturia adversely affects the cardiovascular system by disrupting sleep and causing sleep deprivation^29^. It has been suggested that poor or inadequate sleep may lead to adverse cardiovascular morbidity; however, according to this previous report, nocturia was not associated with cardiovascular disease, including time awake after sleep onset^29^. The report concluded that the relationship between natriuretic peptides, nocturia, and cardiovascular outcomes is complex and requires further study, although nocturia decreases the circulating blood volume and may reduce cardiac preload in patients^29^. NPs also have multifactorial etiologies as well as urologic diseases. Namely, untreated diabetes, insomnia, cardiovascular disease, chronic kidney disease (CKD), and primary polydipsia have been identified^30, 31^. The present study also investigated various factors such as diabetes, hypertension, insomnia, and CKD; however, these factors were not detected as significant risk factors for NP. However, diabetes and hypertension are known risk factors for ischemic heart disease and cerebrovascular disease^32–34^. In addition, obstructive sleep apnea is known to be a risk factor for cerebrovascular disease^35^. The results of this study suggested that ischemic heart disease and cerebrovascular disease might be associated with a higher risk of developing vascular disease compared to diabetes and hypertension, and might also contribute to the occurrence of NP.

Several limitations remain to be addressed in this study. First, this was a retrospective, multicenter study; thus, the potential for inherent bias could not be eliminated. Second, nocturia and NP were examined using bladder diaries recorded by patients, suggesting that the criteria for these assessments may not be consistent. Additionally, some patients may not have accurately recorded urine volume and the frequency of voiding in their bladder diaries. Third, there was a need to examine the validity of the cutoff value of NPi for the diagnosis of NP, which was adopted in this study. The definition of NPi as > 33% was commonly used for patients over 65 years of age, while a definition of 20% was accepted for younger patients^2, 36^. However, a clear definition of NP based on specific age categories has not yet been established. In this study, we adopted NPi > 33% as the definition of NP because the majority of the enrolled patients were ≥ 65 years of age, suggesting that a clear definition of NP based on age categories may be necessary in the future. Fourth, the high proportion of male patients in this study may require cautious interpretation of the results obtained in this study even though nocturia is generally considered to occur regardless of gender^2, 3^. Fifth, the health status of the enrolled patients was not objectively assessed using a screening tool for older adults; therefore, the true impact of aging is unclear. Finally, we were unable to investigate the etiology of NP because we did not perform hematological tests for nocturia or NP.

## Conclusions

To the best of our knowledge, this is the first large, multicenter study to investigate the prevalence of NP in Japanese patients with nocturia. The prevalence of NP in Japanese patients with nocturia is 67.4%. Independent predictors of NP include age ≥ 78 years, BMI < 23.0 kg/m^2^, and the presence of ischemic heart disease and cerebrovascular disease. Whether these factors truly influence NP would need to be examined in future prospective studies with a large number of patients.

## Data Availability

The data that support the findings of this study are available in this manuscript.
